# A simplified bacterial community found within the epidermis than at the epidermal surface of atopic dermatitis patients and healthy controls

**DOI:** 10.1186/s12866-023-03012-7

**Published:** 2023-09-29

**Authors:** Christopher J. Barnes, Maria Asplund, Maja-Lisa Clausen, Linett Rasmussen, Caroline Meyer Olesen, Yasemin Topal Yüksel, Paal Skytt Andersen, Thomas Litman, Kim Holmstrøm, Lene Bay, Blaine Gabriel Fritz, Thomas Bjarnsholt, Tove Agner, Anders Johannes Hansen

**Affiliations:** 1 The Globe Institute, Faculty of Health, University of Copenhagen, Copenhagen K, 1350 Denmark; 2 Department of Agroecology, Faculty of Technical Sciences, Aarhus University, Forsøgsvej 1, Slagelse, 4200 Denmark; 3 Department of Dermatology, Bispebjerg Hospital, University of Copenhagen, Copenhagen, Denmark; 4 Department of Bacteria, Parasites and Fungi, Statens Serum Insitute, Copenhagen, Denmark; 5 Department of Immunology and Microbiology, LEO Foundation Skin Immunology Research Center, University of Copenhagen, Copenhagen, Denmark; 6 Explorative Biology and Bioinformatics, LEO Pharma A/S, Ballerup, Denmark; 7 Bioneer A/S, Kogle Allé 2, Hørsholm, Denmark; 8 Costerton Biofilm Center, Department of Immunology and Microbiology, University of Copenhagen, Copenhagen, Denmark

**Keywords:** Atopic dermatitis, Epidermis, Bacteria, Skin microbiome, Fluorescent in situ hybridisation, *Staphylococcus aureus*

## Abstract

There has been considerable research into the understanding of the healthy skin microbiome. Similarly, there is also a considerable body of research into whether specific microbes contribute to skin disorders, with atopic dermatitis (AD) routinely linked to increased *Staphylococcus aureus (S. aureus)* colonisation. In this study, the epidermal surface of participants was sampled using swabs, while serial tape-stripping (35 tapes) was performed to sample through the stratum corneum. Samples were taken from AD patients and healthy controls, and the bacterial communities were profiled by metabarcoding the universal V3-V4 16S rRNA region. Results show that the majority of bacterial richness is located within the outermost layers of the stratum corneum, however there were many taxa that were found almost exclusively at the very outermost layer of the epidermis. We therefore hypothesise that tape-stripping can be performed to investigate the ‘core microbiome’ of participants by removing environmental contaminants. Interestingly, significant community variation between AD patients and healthy controls was only observable at the epidermal surface, yet a number of individual taxa were found to consistently differ with AD status across the entire epidermis (i.e. both the epidermal surface and within the epidermis). Sampling strategy could therefore be tailored dependent on the hypothesis, with sampling for forensic applications best performed using surface swabs and outer tapes, while profiling sub-surface communities may better reflect host genome and immunological status.

**Supplementary Information:**

The online version contains supplementary material available at 10.1186/s12866-023-03012-7.

## Introduction

A healthy microbiome can prevent pathogen colonisation [1] and promote beneficial immune responses [2]. Consequently, there has been considerable interest in the composition and variation of the skin microbiome [3]. Many studies have found great inter-individual variation in the skin microbiome [4], but also between different parts of the body [5]. The skin can be partitioned into sebaceous (oily), moist and dry habitats, and the bacterial communities of these are well-established and distinct [5]. However, the skin comprises multiple layers; the epidermal layer comprising the outermost stratum corneum, the dermis below, and a layer of subcutaneous fat, each of which have been shown to have distinct bacterial assemblages [6]. The structure of the skin is further complicated by the hair follicles, which penetrate from the dermis to the surface of the epidermis. These have also been found to have discrete bacterial assemblages [7, 8], and have been hypothesised to serve as a reservoir, allowing commensal skin microbes to repopulate the skin after disturbances [8]. However, the vast majority of studies investigating the skin microbiome have used swabs that only sample the bacteria of the very outer surface of the epidermis [9]. Therefore, many questions remain regarding the distribution and function of the bacteria beneath this layer. We hypothesise that these sub-surface bacterial communities will better represent the active component of the host’s skin microbiome since they are less likely to be highly transient environmental contaminants. We will test this by comparing the bacterial communities across the epidermis, predicting that the bacterial communities taken from beneath the surface will sample a ‘core’ bacterial community that better separates individuals.

Disturbances in the skin microbiome are associated with disease, such as the colonisation of *Staphylococcus aureus (S. aureus)* and *Streptococcus pyogenes* (*S. pyogenes*) causing skin infections [10]. Atopic dermatitis (AD) is a common, chronic skin disorder [11, 12] that is associated with a perturbation of the skin microbiome (dysbiosis) [13], but also with genetic mutations within hosts, such as a mutation within the filaggrin gene [14]. Generally, the skin of AD patients has been shown to have reduced bacterial diversity and increased *S. aureus* colonisation compared to healthy control skin [15]. Similarly, the lesional skin has also been shown to differ from both the unaffected skin of the same patient, and from healthy control skin [15]. These characterisations of the skin bacteria were almost exclusively made by sampling the outer epidermis, and it is not known whether the lower skin layers are also perturbed with AD. While certain strains of *S. aureus* were shown to induce lesions in mice [1], in humans, it remains unknown whether *S. aureus* drives AD pathogenesis or opportunistically colonises the skin of AD patients. If this dysbiosis associated with AD is detectable deeper within the skin, it is further indicative that the microbiome has a deterministic role within AD pathogenesis. In this work, we investigated whether the bacterial dysbiosis associated with AD was more clearly detected within the lower epidermal samples through the removal environmental contaminants.

In this project we have two main hypothesises, with hypothesis 1 split into 3 sub-hypotheses. In *hypothesis 1a* we propose that the inner epidermal skin bacteria significantly differ from the outer (that are normally sampled in other studies), with significantly higher bacterial richness at the epidermal surface. We further hypothesise (*hypothesis 1b*) that the outer epidermis will contain a number of bacteria that are likely environmental contaminants (i.e. bacteria found exclusively at the surface), which likely superficially interact with the skin. In order to test these, the bacterial communities were sampled from the elbow crease (semi-moist habitat), volar forearms (dry habitat) from AD-patients and healthy controls, and from lesions (AD-patients only) of participants, which were profiled by metabarcoding of the 16S rRNA universal bacterial regions (complete amplicon sequence variants [ASV] in Table S1). At each location, swabbing was performed to sample the surface. Then, serial tape-stripping was performed 35 times at a single location, in order to penetrate through the stratum corneum and deep into the epidermis (with tape 35 approximately 33% through the epidermis; Table S2) [16]. Using individual tape samples, we found significant differences between the outer and inner epidermal communities, with a number of bacteria found disproportionately at the epidermal surface. We therefore further hypothesised that the inner epidermal bacteria will have a more similar bacterial assemblage to the dermis (*hypothesise 1c*) than the outer epidermis, and can therefore be used as an alternative to sample the inner skin microbiota without invasive biopsies, to ultimately improve patient participation within sub-surface skin microbiome studies. To test this, we compared our epidermal samples to a pre-existing dataset of comparable dermal communities that were sampled using biopsies [6].

In *hypothesis 2*, we propose that the dysbiosis associated with AD is detectable across the epidermis, and not only at the outermost epidermal layers that are routinely sampled. We further hypothesise that repeated tape-stripping will remove putative environmental contaminants to better delineate the bacterial communities of AD patients and healthy controls. This was performed by comparing the bacterial communities of AD patients and healthy controls at different layers of the epidermis.

## Results

### Hypothesis 1a—Richness significantly declines from the surface of the epidermis

**Fig. 1:**
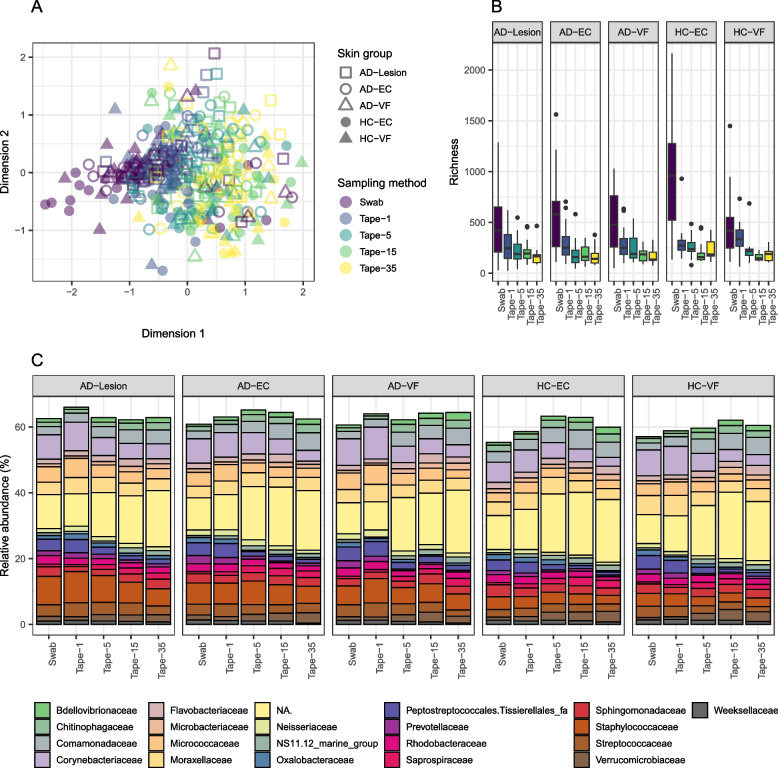
**A** Non-metric multidimensional scaling of the bacterial community, with colour representing the sampling method and colour representing the skin group (a combination of body location and AD status). **B** ASV richness between sampling methods and skin groups. **C** Average taxonomic composition at family level for each skin group for each sampling strategy. Families of less than 1% mean relative abundance were filtered for readability, while the NA category represents ASVs unassigned to a family

Swabs were used to sample the epidermal surface, while serial tape-stripping was used to penetrate from the surface through the stratum corneum and further into the epidermis (including healthy controls and AD samples) [16]. There were significant differences in the bacterial community composition between epidermal layers, accounting for a total 3.6% of community variation (Table 1; Fig. 1A). Pairwise comparisons revealed two distinct bacterial communities, one at the epidermal surface (swab and tape-1 communities), and one in the epidermis (tapes-15 and 35 communities) (Table S3), while Tape-5 s fall between the two. In terms of ASV richness, it was significantly lower within the epidermis than at the epidermal surface. However, pairwise analyses revealed that ASV richness declined as a gradient, as opposed to the two distinct groups found in community composition. The surface swabs being the most ASV rich (596.9 ± 435.0 ASVs) (Fig. 1B; Fig. S1), followed by tape 1 samples, (301.9 ± 166.5 ASVs), tape-5 (230.9 ± 131.7 ASVs), tape-15 (190.4 ± 90.3 ASVs) and tape-35 (184.9 ± 86.6 ASVs). When visualised at the family level, all samples were dominated by typical skin bacteria. However there were consist differences visible across the epidermis, with for example, decreasing abundance of Corynebacteriaceae observed in all skin groups (Fig. 1C). Mixed linear modelling revealed all abundant families (< 1% mean relative abundance) significantly differed over skin depths (Table S4), with the exception of the Sphingomonadaceae. Interestingly, families showed large changes between the swabs and outer tapes (1 and 5), while remaining relatively stable relative abundances between Tapes 15 and 35 (Fig. S2).

**Table 1:** Using serial tape-strips and swabs from healthy controls and AD patients, inter-individual variation within the bacterial skin community was analysed using PERMANOVA, while differences in ASV richness was analysed using generalised linear modelling and likelihood ratio testing (chi-squared). Differences in community composition and ASV richness associated with skin depth and skin group (a combination of body location and AD status) were performed using PERMANOVA and mixed linear modelling while accounting for inter-individual variation

Composition				ASV Richness			
Variable	F-model	R2	*P*-value	Variable	AIC	LRT	*P*-value
Inter-individual variation	**2.4404**	**0.199**	**0.001**	Inter-individual variation	**30766178**	**4296.7**	**0.000**
Skin depth	**3.4632**	**0.036**	**0.001**	Skin depth	**30766178**	**4296.7**	**0.000**
Skin group	**1.6928**	**0.018**	**0.001**	Skin group	**30766178**	**4296.7**	**0.031**

### Hypothesis 1b – Many bacteria were disproportionately located at the epidermal surface

**Fig. 2:**
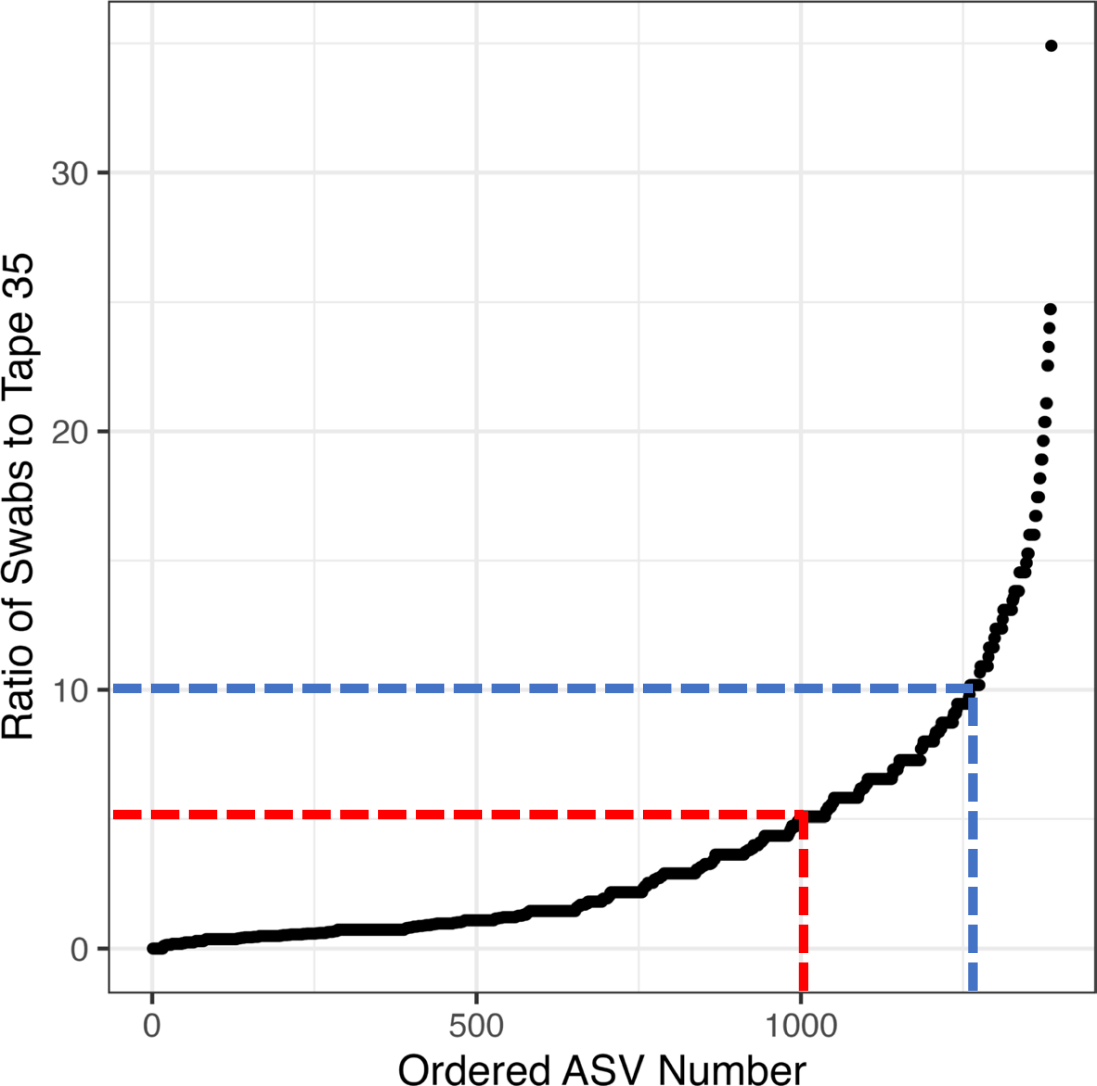
The percentage of samples an ASV was present in for the swabs was then divided by the percentage of samples it was found within the tape 35 samples (y-axis). These were ordered by size (x-axis) and plotted. The red line represents ASVs that are 5 times more frequent within the swab samples than the tape 35 (386 ASVs), and the blue represents 10 times (126 ASVs). Not shown was 409 ASVs that were exclusively found in the swabs, while only 7 were found in the tape 35 samples and not swabs

**Fig. 3:**
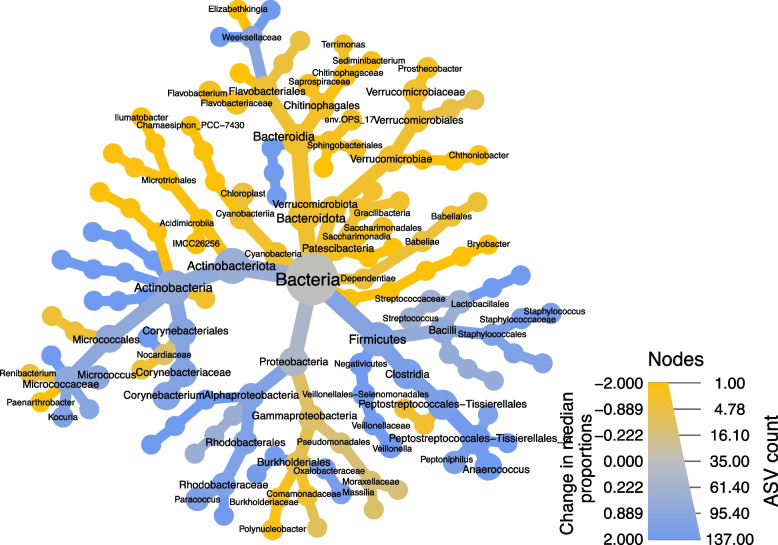
Significant differences in ASV abundance across the epidermis was analysed using mixed linear modelling (AD patients and healthy participants visualised together). A heat tree was constructed from the 238 ASVs that were found to significantly vary between tapes, with node size representing the total number of ASVs (*q*-value > 0.05) and node colour representing the change in ASV abundance (log_2_) between the swabs (higher relative abundance in swabs represented by blue coloration) and the tape-35 samples (higher relative abundance in tape 35 represented by green coloration)

With fewer ASVs found deeper into the stratum corneum (i.e. lower ASV richness in Tapes-5 to 35 samples), the distribution of individual taxa was explored to determine whether they were found disproportionately at the epidermal surface. The persistence of each ASV was calculated for each layer (i.e. the percentage of samples an ASV was detected in). Initially, the persistence of each ASV within swabs (the outermost layer) was divided by the persistence within the tape 35 samples (the innermost). In total, there were 1795 ASVs found in one or both of these sample sets, and 409 found exclusively at the surface, while only 7 were found exclusively within the tape 35 samples. Additionally, there were 386 more ASVs (of the total 1795 ASVs) that were × 5 more common in the swabs than the tape 35 samples, with 126 of these × 10 more common (Fig. 2). A similar pattern was observed when comparing the tape 1 and tape 35 samples, ruling out methodological variation determining these differences in persistence. Mixed linear modelling was also used to correlate individual ASV abundance (with a mean relative abundance greater than 0.5%, a total of 387 ASVs) against depth, while accounting for inter-individual variation and body locations. A total of 238 ASVs that significantly varied after correction for multiple comparisons (q-value > 0.05) (Table S5). Of these 238 ASVs, 118 ASVs increased as we penetrated through the stratum corneum, while 120 ASVs decreased (Fig. 3). These 238 ASVs were visualised within a heat tree, revealing that Actinobacteria, Firmicutes and Alphaproteobacteria were higher at the epidermal surface (blue on the heat tree), while the Gammaproteobacteria, Verrucomicrobiota, Bacteroidota and Patescibacteria increased in relative abundance within the epidermis (green on the heat tree) (Fig. 3). It should be emphasised that these are relative abundances, not absolute (i.e. total bacterial abundance is very likely to be less within the epidermis than at the epidermal surface).

As an additional quality assurance test of sampling method bias, differences in the abundance of abundant ASVs were compared between the swabs and tape-1 samples (only ASVs with a mean relative abundance above 0.5%; Fig. S3). Here, only 7 individual ASVs significantly differed in relative abundance (257 ASVs were tested) (Table S6), and while not inconsequential, was much smaller than the number of ASVs that differed across the epidermis.

### Hypothesis 1c—The dermis has a distinct bacterial assembly from the epidermis

As the lower epidermal communities were found to be distinct from the epidermal surface, we further explored whether the lower communities of the epidermis were becoming more similar to the communities of the dermis, or whether the bacteria of the dermis form their own distinct assembly. Previous biopsies that were partitioned into epidermal and dermal compartments were compared to the swabs and tapes from this study [6]. The dermal communities were significantly different in community composition from all epidermal communities, although they were most similar to the epidermal communities produced from the same study (Fig. S4A; Table S7). The dermal samples had significantly lower ASV richness than all epidermal samples, while the corresponding epidermal samples from the biopsies did not (with the exception of the Tape-1 samples) (Fig. S4B; Table S7). Specifically, the dermis was distinguished from the epidermal samples by higher relative abundances of the Streptococcaceae and Moraxellaceae families (Fig. S4C).

### Objective 2—The dysbiosis associated with AD was detectable primarily at the epidermal surface

There were significant differences in the bacterial community composition and ASV richness between the AD patients and healthy controls when the epidermal layers were analysed together (Table 1; Table S8). We further explored whether the dysbiosis associated with AD was detectable throughout the epidermis by comparing AD samples to healthy controls at each skin depth independently. Skin group (volar forearms, elbow crease and lesions from AD patients, volar forearms and elbow creases from healthy controls) was correlated against the communities of each skin layer independently (surface, tape-1, tape-5, tape-15 and tape-35). The bacterial communities sampled at the epidermal surface and tape-5 also correlated by skin group (Table 2), accounting for 5–6% of community variation, but there was no significant variation at the lower skin depths. ASV richness was correlated against skin group while accounting for inter-individual variation (Table 2). At the epidermal surface, ASV richness significantly varied between skin groups from the swabs, being significantly higher in elbow creases of healthy patients (947.1 ± 594.0) and AD patients (607.9 ± 594.0) compared to the volar forearms (healthy control: 480.1 ± 335.8 and AD: 498.0 ± 305.2). Lesions had the lowest ASV richness, at 471.6 ASVs (± 321.4).

**Table 2:** Samples were partitioned by sampling strategy (swabs, different tape depths) and the bacterial composition was analysed against inter-individual variation, sampling location, skin status and skin group individually using PERMANOVA. Similarly, differences in ASV richness associated with explanatory variables was analysed for each sampling strategy separately using mixed linear modelling

**Composition**			
**Skin depth**	**Parameter**	**Inter-individual variation**	**Skin group**
**Swabs**	F-model	**2.187**	**1.111**
	R2	**0.595**	**0.051**
	*P*-value	**0.001**	**0.001**
**Tape-1**	F-model	**1.726**	1.12
	R2	**0.552**	0.053
	*P*-value	**0.001**	0.28
**Tape-5**	F-model	**1.284**	**1.095**
	R2	**0.541**	**0.061**
	*P*-value	**0.001**	**0.018**
**Tape-15**	F-model	**1.197**	1.056
	R2	**0.531**	0.06
	*P*-value	**0.001**	0.345
**Tape-35**	F-model	**1.147**	1.025
	R2	**0.558**	0.065
	*P*-value	**0.001**	0.416

**Richness**			
**Skin depth**	**Parameter**	**Inter-individual variation**	**Skin group**
**Swabs**	AIC	**10320270**	**1315.8**
	LRT	**1070.3**	**22.93**
	*P*-value	**0.000**	**0.000**
**Tape-1**	AIC	**2327519**	1106.3
	LRT	**870.5**	2.32
	*P*-value	**0.000**	0.678
**Tape-5**	AIC	1231403	912.1
	LRT	703.78	0.25
	*P*-value	0.2733	0.8836
**Tape-15**	AIC	**571365**	846
	LRT	**640.51**	2.77
	*P*-value	**0.023**	0.597
**Tape-35**	AIC	**472461**	757.3
	LRT	**572.04**	3.97
	*P*-value	**0.044**	0.41

Filaggrin mutations [14, 17] and steroid [18, 19] use have previously been linked with variation in skin bacteria. The effects of each were explored on bacterial richness and community composition using samples from AD patients only. We found small significant differences in community composition associated with both filaggrin mutations (Table S9; Fig. S5) and steroid use (Table S10; Fig. S6), but no effects on bacterial richness of either.

The effect of AD was also explored on the *Staphylococcus* community since the genus has been shown to increase significantly with AD [14]. There were significant differences in relative abundance of *Staphylococcus* (Fig. S7A; Table S11), but not richness differences (Fig. S7B; Table S11). Similarly, there was a significant effect of AD on the community composition on the combined and individual communities (with the exception of tape-35), with AD explaining a larger percentage of variation of the *Staphylococcus* community compared to the overall bacterial community (Fig. S7C; Table S11).

**Fig. 4:**
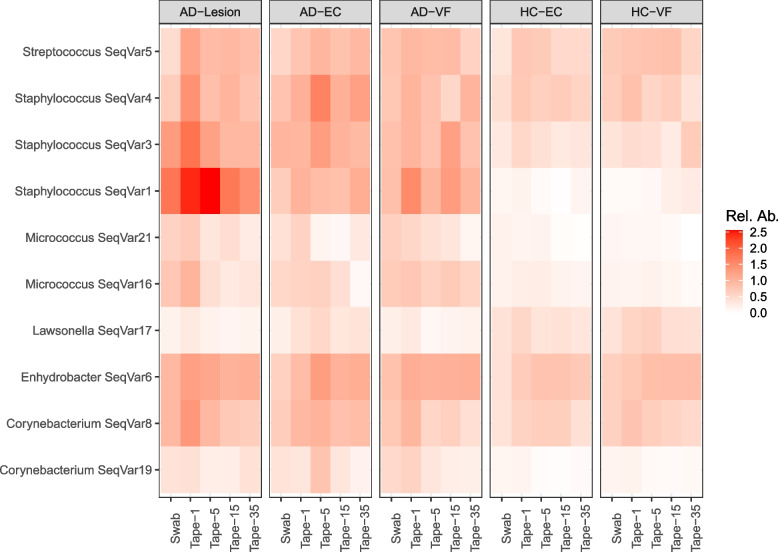
Heatmap of ASVs that significantly varied between skin groups, which were visualised across the epidermis. *Staphylococcus* ASVs 1, 3 and 4 were 3 of the most abundant ASVs that were in highest abundance within the lesions of AD patients, followed by the non-lesional skin of AD patients, then the healthy controls

The individual ASVs that distinguished skin groups were investigated. There were 12 ASVs that varied between skin groups, including *Staphylococcus* ASVs 1, 3 and 4 (Fig. 4). These were three of the four most abundant ASVs across the dataset that were most abundant in the lesional samples, followed by the non-lesional samples and then healthy controls. There was only one ASV that was more persistent in the healthy controls than in AD samples, a Lawsonella ASV (ASV17). Importantly, these ASVs showed consistent differences between skin types across skin depths (e.g. *Staphylococcus* ASV1 was enriched in swabs and all tapes from AD patients compared to all samples from healthy controls).

## Discussion

### Hypothesis 1

In this work, our initial hypothesis was that the bacteria of the inner epidermis differ from the outer (non-randomly spatially structured). Here, we found a significant reduction in ASV richness as we penetrated through the epidermis. This was driven by a number of ASVs that were disproportionately found at the epidermal surface, and not within the inner epidermal samples. These ASVs may represent environmental contaminants, although further analysis of their functioning is required to confirm they do not interact in a biologically meaningful way with the epidermis. These inner epidermal communities were a predictable subset of the outer, mirroring previous findings in which the dermis was a subset of the epidermis [6]. However, comparisons between our data and the previously generated dermal communities suggest that the bacteria of dermis are an alternative subset of the epidermal surface communities. Both the epidermis and dermis have been shown to uptake atmospheric oxygen [20], however there is an oxygen gradient from the surface to the dermal layer. Interestingly, the Corynebacteriaceae (3.8%) and Peptostreptococcales (2.6%) underwent the largest decline in mean relative abundance through the stratum corneum, with the former being aerobic microbes while the latter being anaerobic. Meanwhile the Verrucomicrobiaceae and Comamonadaceae families underwent significant increases in the inner epidermal samples compared to the surface, both of which are aerobic microbes. This suggests that oxygen is not determining differences in the epidermal samples, but may still play a role in differentiating the epidermal and dermal communities [6]. Ultimately, serial tape-stripping cannot be used as an alternative to biopsies to sample the dermal-like microbial communities, but can be used to access a representative version of the epidermal surface with lower complexity.

### Hypothesis 2

In addition to comparing the communities across the epidermis, we also determined whether AD affected the bacterial communities throughout the stratum corneum, since previous investigations have nearly exclusively sampled the epidermal surface only. While a significant difference in bacterial communities was found between AD patients and healthy controls, differences at the overall community level were limited to the upper stratum corneum/epidermis. However, a number of ASVs that distinguished skin groups were consistently found to differ across the epidermal surface. Differences in the skin microbiota associated with AD have been observed beneath the epidermal surface, with for example S*. aureus* detected within the dermis of AD patients. However, to the best of our knowledge, no study to date has looked at the bacterial community composition below the epidermal surface. The identification of a number of non-*Staphylococcus* ASVs that increased in abundance with AD, alongside the *Lawsonella* ASV17 which was reduced in abundance within AD patients, suggesting that serial tape-stripping could help identify complex microbe-microbe interactions that may influence AD pathogenesis by reducing the complexity of the microbial community.

*Tape-stripping allows access to sub-dermal microbial communities in future studies, which may better reflect the host’s core microbiome*. Inter-individual variation was by far the most strongly associated factor with both the community composition and ASV richness across the stratum corneum [4]. The differences in the bacterial community between layers were primarily linked to less abundant ASVs. Thus, there was a shared core microbiome within patients that was persistent between skin depths and body locations, and this also strongly differentiated individuals. This was even conserved across both the lesional and non-lesional skin of AD patients. Serial tape-stripping reduced the number of rare and low abundance ASVs, while the differences in bacterial communities associated with inter-individual variation and lesioning were retained. Using the 15^th^ tape of the serial stripping protocol may therefore better sample the hypothesised ‘core’ microbiome without the need for invasive biopsies [21], which may increase participation in such studies. Furthermore, repeated tape-stripping may provide better fine-scale structure of the epidermis since biopsies compromise the structure of the epidermis, dermis and subcutaneous tissue [22]. Therefore, future studies could benefit by tailoring their sampling strategy to their hypotheses, with for example, sampling for forensic applications best performed using surface swabs [23], while profiling sub-surface communities may better reflect host genome and immunological status [2]. This is particularly interesting as the bacteria [23] and immune status [24] of AD patients have independently been identified to form distinct clusters, but the relationship between the two is unknown, but may represent multiple different mechanisms behind AD pathogenesis. Within the contexts of AD, serial tape-stripping could be used to explore whether the regulation of epidermal bacteria might differ between layers. For example, the outer epidermis might be affected to a greater extent by the skin barrier being drier during AD flareups [24]. Meanwhile, differences associated with AD found within the lower epidermal layers might reflect more longer-term differences in host immunology [25]. Further investigations with larger sampling numbers using the tape-stripping method would allow for this to be explored more comprehensively.

### Study limitations

While significant differences in the bacterial community composition were observed between AD samples and healthy controls, differences were not as large as expected. Similarly, the expected decline in bacterial richness associated with AD was not found. While greater differences were found when studying only the *Staphylococcus* community, they were still relatively small compared to other methods such as culturing. This is likely due to the limited ability of metabarcoding to separate closely related taxa, including different *Staphylococcus* species and strains. Further, the genus *Cutibacterium* are abundant within the skin microbiome, but were also underrepresented in our samples (means of ~ 1.0 ASV per sample, ~ 0.3% relative abundance) [26]. An, untargeted approach like shotgun metagenomics reduces these biases and can even identify strain variants (of more and less pathogenic *S. aureus*). However, this approach too will be challenging to profile sub-surface skin microbiomes since untargeted analyses are dominated by human DNA (unpublished data). Treatments are possible to enrich the proportion of microbial DNA relative to the host [27, 28], however, they add further costs to a method that is already notably more expensive than metabarcoding. One promising alternative would be the use of ultra-long amplicons, targeting the whole 16S rRNA region, which could represent a cost-effective approach to sequencing large numbers of samples and providing high levels of taxonomic resolution [29].

## Conclusions

This study provides novel insights into the distribution and composition of the skin bacteria, showing that many bacteria are disproportionately located at the surface of the epidermis and are likely environmental contaminants. This emphasises the importance of sampling depth and sampling techniques in future studies of the skin microbiome in AD, and the potential of utilising tape-stripping to investigate the ‘core microbiome’ without more invasive techniques such as biopsies [6]. Interestingly, the dysbiosis of the bacteria associated with AD was larger at the epidermal surface at the community level. Despite this, a number of ASVs were found to consistently differ with AD status, suggesting that serial tape-stripping could aid in identifying taxa beyond *S. aureus* that influence AD pathogenesis. Additional experiments using the approach to sample more severe AD patients over flareups could be particularly informative [30].

## Methods and methods

### Participants

Participants within this study were recruited from the outpatient clinic of the Department of Dermatology, Bispebjerg Hospital, with samples taken from March-December 2017. AD patients were identified according to the UK-criteria, and were all over 18 years of age (for legal consent) and under the age of 60 (to avoid confounding age effects) [31]. Patients that had undergone phototherapy or systemic immunosuppressive drugs within the last four weeks prior to sampling were excluded. Patients were instructed not to use topical corticosteroids 7 days prior to sampling.

In total 19 AD patients (7 men, 13 women) were recruited alongside 19 healthy controls (12 men, 8 women), ranging from between 19 and 59 years of age (full metadata available as Table S2). Objective Scoring of Atopic Dermatitis (O-SCORAD) was used to quantify AD severity (mean 20.5 ± 7.7) [32], while all participants underwent a screening for filaggrin mutations within the R501X, 2282del4 and R2447X genes [14].

### Sampling of tape strips

For 19 healthy controls and 19 AD patients, the volar forearm and elbow crease were sampled (areas without any sign of lesions), while a lesion was also sampled for the AD patients. Lesions from AD patients were sampled from the volar forearms (10 patients), however other dry skin lesions were targeted in their absence (abdomen, back, foot and leg), which have previously found to not differ in bacteria between body locations [4]. At each sample site, swabs and tape-strips were collected [16, 33], and for comparability, tape-stripping was performed on skin that was adjacent to the swabbing site to avoid sampling an already perturbed site. Tape-stripping consisted of 35 consecutive adhesive D-Squame tapes (22 mm diameter; CuDerm, Dallas TX, USA) that were placed onto the skin and pushed for 10 s using a standardized pressure device (225 g cm^−2^, D-Squame Pressure Instrument D500). Tape-stripping was stopped at tape-35 as at this point, the stratum corneum was entirely penetrated and patients would begin to bleed [16]. Each tape strip was placed into separate cryo-vials and immediately stored at –80 °C.

### DNA Extraction

Swabs and tapes underwent DNA extraction following the DNeasy Blood and Tissue Kit (QIAGEN, Germany) manufacturer’s instructions, but with a few modifications. It was not possible to extract every tape, however, the initial tapes remove more of the stratum corneum than the later tapes [16]. Therefore, we processed more of the initial tapes (tapes-1, 5 and 15) than the later tapes (tape-35). Initially, samples were placed in a lysing matrix E column (MP Biomedicals, Cambridge, UK) with 600 µL of ATL buffer, then underwent mechanical lysis within a tissue lyser (two periods of shaking at 30 Hz s^−1^ for 30 s using a tissue lyser; TissueLyser II, QIAGEN, Denmark). The supernatant was transferred to a 2 mL tube and 20 µL of proteinase K was added before incubation at 56 °C overnight. The standard protocol was then followed, with the addition of a 15 min incubation period at 37 °C with elution buffer within the spin column before the final centrifugation step. Extractions were performed in batches of 31 samples with an extraction negative.

### Sequencing preparation and bioinformatic analysis

Bacterial communities were characterised by metabarcoding the universal V3-V4 16S rRNA region of bacteria, using the 341F (5’-CCTAYGGGRBGCASCAG-3’) and 806R (5’-GGACTACNNGGGTATCTAAT-3’) primers. Thermocycler conditions, library creation (using TruSeq DNA PCR-Free Library Preparation Kits (Illumina, CA, USA)) and sequencing (on a MiSeq 4000 platform) were performed as per Barnes et al [33]. In total, a combined 68 negatives (extraction blanks and PCR negatives) were included.

Similarly, bioinformatics were performed using DADA2 as per Barnes et al [33], with the ASV approach showing more reproducible results compared to operational taxonomic units [33]. Within the main study (comparing swabs and tapes) a total, 37,629,622 reads were assigned to samples, which accounted for 77,268 reads per sample (487 samples). The datasets generated during the current study are available at NCBI’s Sequence Read Archive (under the BioProject PRJNA1010568). Potential contaminants were removed from samples if they were present in more than a third of the negative controls (extraction negatives or PCR blanks). Additionally, ASVs not assigned to bacteria or archaea were also removed. Finally, reads underwent a fourth-root transformation for normalisation, before being converted to relative abundances for subsequent statistical analyses.

### Re-using biopsy data to compare the bacteria between epidermal layers to the dermal bacteria

For comparisons with the dermis, raw sequencing files from Bay et al. (2020) [6] were downloaded from the NCBI (BioProject accession no. PRJNA510725) and reanalysed with our data using DADA2 and renormalised. These biopsies (mean of 10,121 reads per sample) had far fewer reads on mean average than in this study (mean of 63,947 reads per sample), and therefore all samples were rarefied to 2,000 reads before comparisons were made.

### Statistics

All statistical analyses performed in the statistical computing language *R* and visualised with ggplot2 package [34], with the exception of heat trees that were produced using the Metacoder package [35]. All scripts used in statistical analysis are freely available online (https://github.com/drcjbarnes/epidermal_bacterial_variation). Initially, inter-individual variation of the bacterial community was explored by calculating a Bray–Curtis similarity matrix then performing PERMANOVA on the matrix. Significant differences in ASV richness were analysed using generalised linear modelling (GLM) and significant testing with likelihood ratio testing (Chi-square).

Subsequently, significant differences in community composition and ASV richness associated with skin depth and skin groups were explored using PERMANOVA and mixed linear modelling (MLM) respectively, while accounting for inter-individual variation (which served as the strata/random effect). Additionally, data was partitioned into each skin depth and analysed separately to test for significant inter-individual variation and between skin groups (while controlling for inter-individual variation). Pairwise comparisons were performed on the community data by using the betadisper function to calculate the Euclidean distances from the Bray–Curtis similarity matrices, and Tukey (Honest Significant Differences) tests were performed on these. Similarly, pairwise comparisons in ASV richness were performed by performing Tukey tests.

Differences in the relative abundance of the most abundant individual ASVs (mean relative abundance > 0.5%) within swabs and tape-1 samples were analysed using paired Student’s *t*-tests. Significant variation in the relative abundance of the most abundant ASVs was also explored using mixed linear modelling, with skin depth serving as the fixed effect and participant number as the random effect.

Pearson’s correlations were performed to correlate the abundances and occurrences of ASVs between methodologies, and correlating ASV relative abundance to the mean average number of samples within participants it was detected within.

### Supplementary Information

**Additional file 1:** **Figure S1.** ASV richness within swabs and tape depths, with different skin groups aggregated. **Figure S2.** Nearly every bacterial family varied in relative abundance across the epidermis. Here we found that Tapes-15 and 35 generally contained similar abundances, while the majority of variation at the epidermal surface (from swabs to tape-5 samples). **Figure S3.** (A) Mean relative abundances and (B) occurrences within patients in swabs were plotted against their equivalent within their tape-1 equivalents. Similarly, their number of occurrences within the tapes-1, 5, 15 and 35 from a single body location of a patient was plotted against their mean relative abundance within the same patient’s body location. **Figure S4.** Previously, biopsies were taken from healthy controls and partitioned into epidermal (epidermis) and dermal (dermis) compartments [4], and their bacterial communities characterised. The (A) composition (non-metric multidimensional scaling) and (B) richness of the epidermal and dermal compartments were compared to tapes and swabs from this experiment. (C) The taxonomic community was further explored at family level. **Figure S5.** Filaggrin mutations were identified within AD patients, and differences in the bacterial community composition (B, D) and richness (A, C) were visualised from both non-lesional (A, B) and lesional samples (C, D). **Figure S6.** A subset of AD patients had undergone topical steroid treatments. Therefore, differences in the bacterial community composition (B, D) and richness (A, C) were assessed from both non-lesional (A, B) and lesional (C, D) samples. **Figure S7.** The *Staphylococcus* community was extracted from the bacterial dataset. Differences in (A) *Staphylococcus* relative abundance, (B) richness and (C) community composition were visualised to investigate for differences between skin types.

**Additional file 2:** **Table S1.** Complete ASV table for all samples and negatives. **Table S2.** Complete metadata table for all samples and negatives. **Table S3.** Pairwise comparisons for community composition and ASV richness. **Table S4.** ASVs that were assessed for significant variation in relative abundance across the epidermis. **Table S5.** Significant differences in the relative abundance (%) of bacterial families between epidermal layers was tested for using mixed linear modelling. **Table S6.** Paired t-tests were performed to identify the individual ASVs that were significantly different between surface swabs and tape-1 samples. **Table S7.** Pairwise comparisons for community composition and ASV richness. **Table S8.** Pairwise comparisons for community composition and ASV richness between skin groups. **Table S9.** Significant differences in community composition and ASV richness were tested for between AD patients with and without filaggrin mutations. **Table S10.** Significant differences in community composition and ASV richness were tested for between AD patients that were using topical steroids (none, short, medium and long-term use). **Table S11.** Reads assigned to Staphylococcus ASVs were extracted and underwent analyses for signifcaint community variation, differences in relative abundance and ASV richness.

## Data Availability

Both raw sequencing files and the resulting datasets generated and/or analysed during the current study are available on the NCBI’s Sequence Read Archive (under the BioProject PRJNA1010568). Meanwhile additional supporting information is freely available at the University of Copenhagen’s Electronic Research Data Archive (https://erda.ku.dk/archives/d4617db3c2f65248026ac54e1cb03bba/published-archive.html).
