# Supplementary material for: A simplified bacterial community found within the epidermis than at the epidermal surface of atopic dermatitis patients and healthy controls
Source: BMC Microbiol. 2023 Sep 29;23:273. doi: 10.1186/s12866-023-03012-7 (PMC10540355; doi:10.1186/s12866-023-03012-7)
Supplement: Supplementary file 1 — Additional file 1: Figure S1. ASV richness within swabs and tape depths, with different skin groups aggregated. Figure S2. Nearly every bacterial family varied in relative abundance across the epidermis. Here we found that Tapes-15 and 35 generally contained similar abundances, while the majority of variation at the epidermal surface (from swabs to tape-5 samples). Figure S3. (A) Mean relative abundances and (B) occurrences within patients in swabs were plotted against their equivalent within their tape-1 equivalents. Similarly, their number of occurrences within the tapes-1, 5, 15 and 35 from a single body location of a patient was plotted against their mean relative abundance within the same patient’s body location. Figure S4. Previously, biopsies were taken from healthy controls and partitioned into epidermal (epidermis) and dermal (dermis) compartments [4], and their bacterial communities characterised. The (A) composition (non-metric multidimensional scaling) and (B) richness of the epidermal and dermal compartments were compared to tapes and swabs from this experiment. (C) The taxonomic community was further explored at family level. Figure S5. Filaggrin mutations were identified within AD patients, and differences in the bacterial community composition (B, D) and richness (A, C) were visualised from both non-lesional (A, B) and lesional samples (C, D). Figure S6. A subset of AD patients had undergone topical steroid treatments. Therefore, differences in the bacterial community composition (B, D) and richness (A, C) were assessed from both non-lesional (A, B) and lesional (C, D) samples. Figure S7. The Staphylococcus community was extracted from the bacterial dataset. Differences in (A) Staphylococcus relative abundance, (B) richness and (C) community composition were visualised to investigate for differences between skin types. [file 12866_2023_3012_MOESM1_ESM.docx]

**Supplementary Figures**


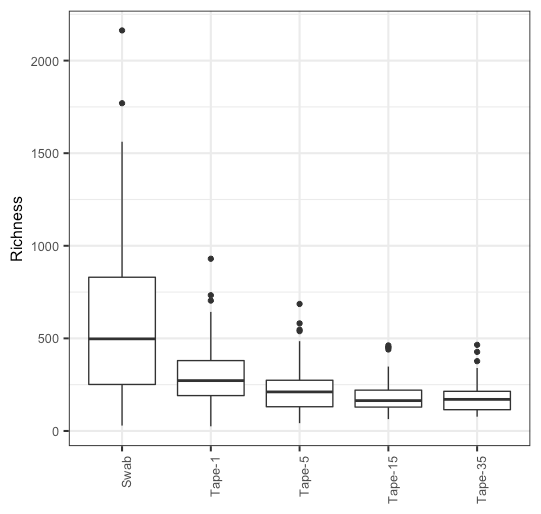


**Figure S1** ASV richness within swabs and tape depths, with different skin groups aggregated.

**Figure S2** Nearly every bacterial family varied in relative abundance across the epidermis. Here we found that Tapes-15 and 35 generally contained similar abundances, while the majority of variation at the epidermal surface (from swabs to tape-5 samples).


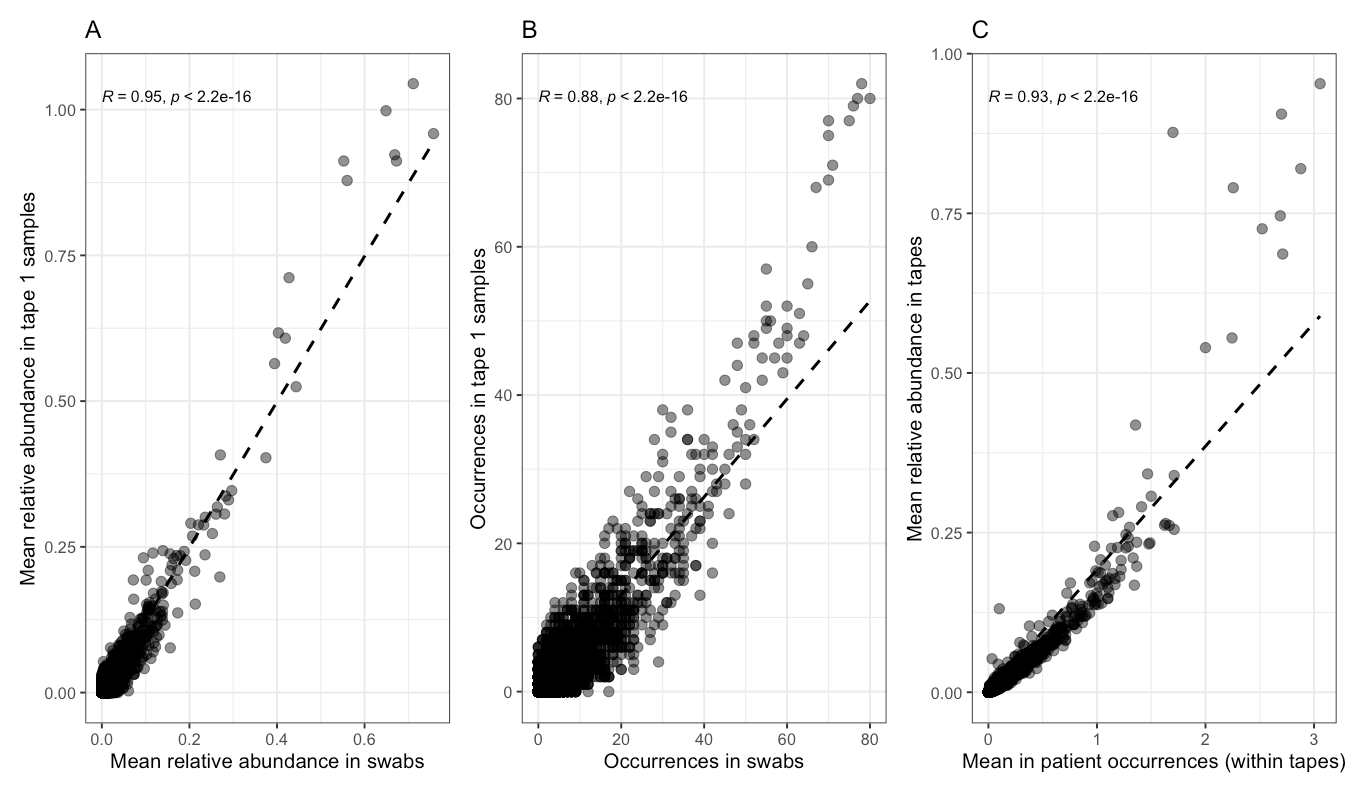


**Figure S3** (**A**) Mean relative abundances and (**B**) occurrences within patients in swabs were plotted against their equivalent within their tape-1 equivalents. Similarly, their number of occurrences within the tapes-1, 5, 15 and 35 from a single body location of a patient was plotted against their mean relative abundance within the same patient’s body location.


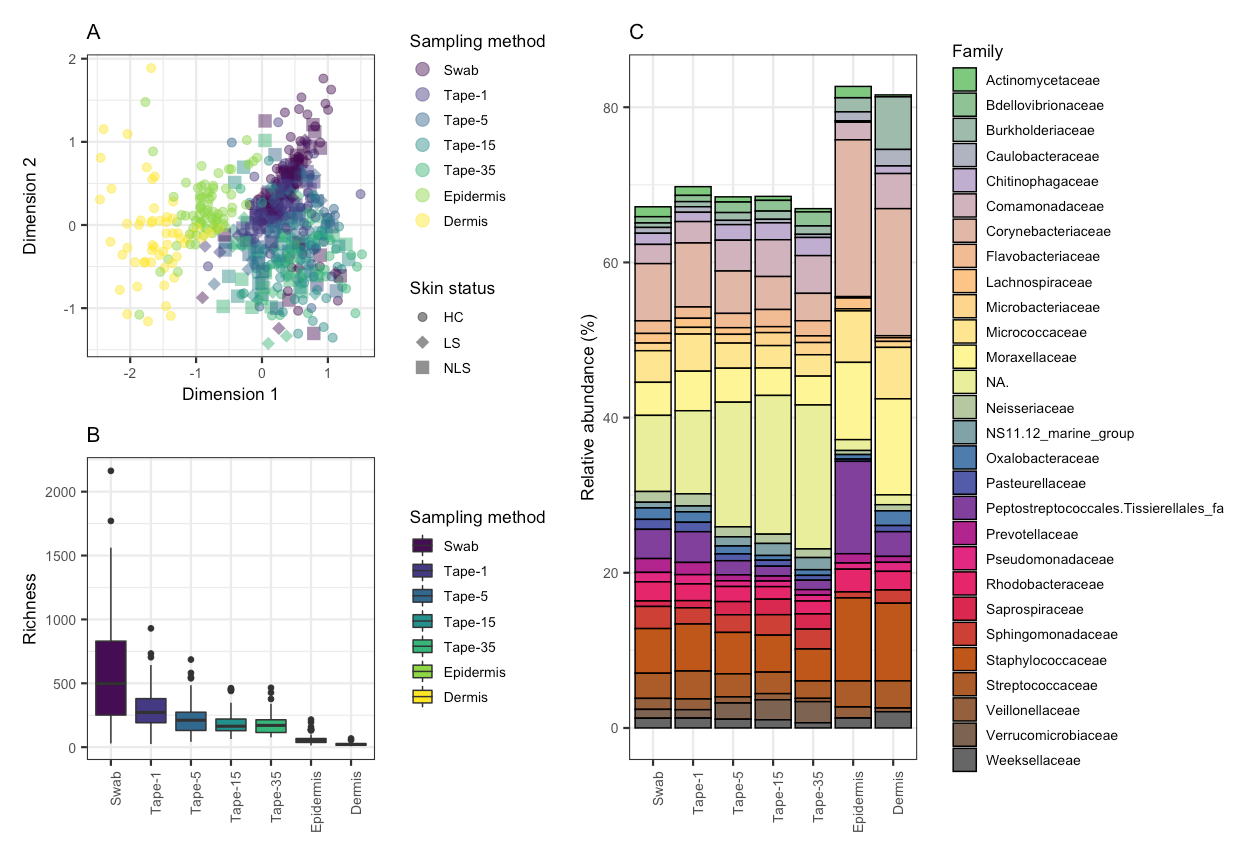


**Figure S4** Previously, biopsies were taken from healthy controls and partitioned into epidermal (epidermis) and dermal (dermis) compartments^4^, and their bacterial communities characterised. The (**A**) composition (non-metric multidimensional scaling) and (**B**) richness of the epidermal and dermal compartments were compared to tapes and swabs from this experiment. (**C**) The taxonomic community was further explored at family level.

**Figure S5** Filaggrin mutations were identified within AD patients, and differences in the bacterial community composition (B, D) and richness (A, C) were visualised from both non-lesional (A, B) and lesional samples (C, D).

**Figure S6** Steroid. A subset of AD patients had undergone topical steroid treatments. Therefore, differences in the bacterial community composition (B, D) and richness (A, C) were assessed from both non-lesional (A, B) and lesional (C, D) samples.


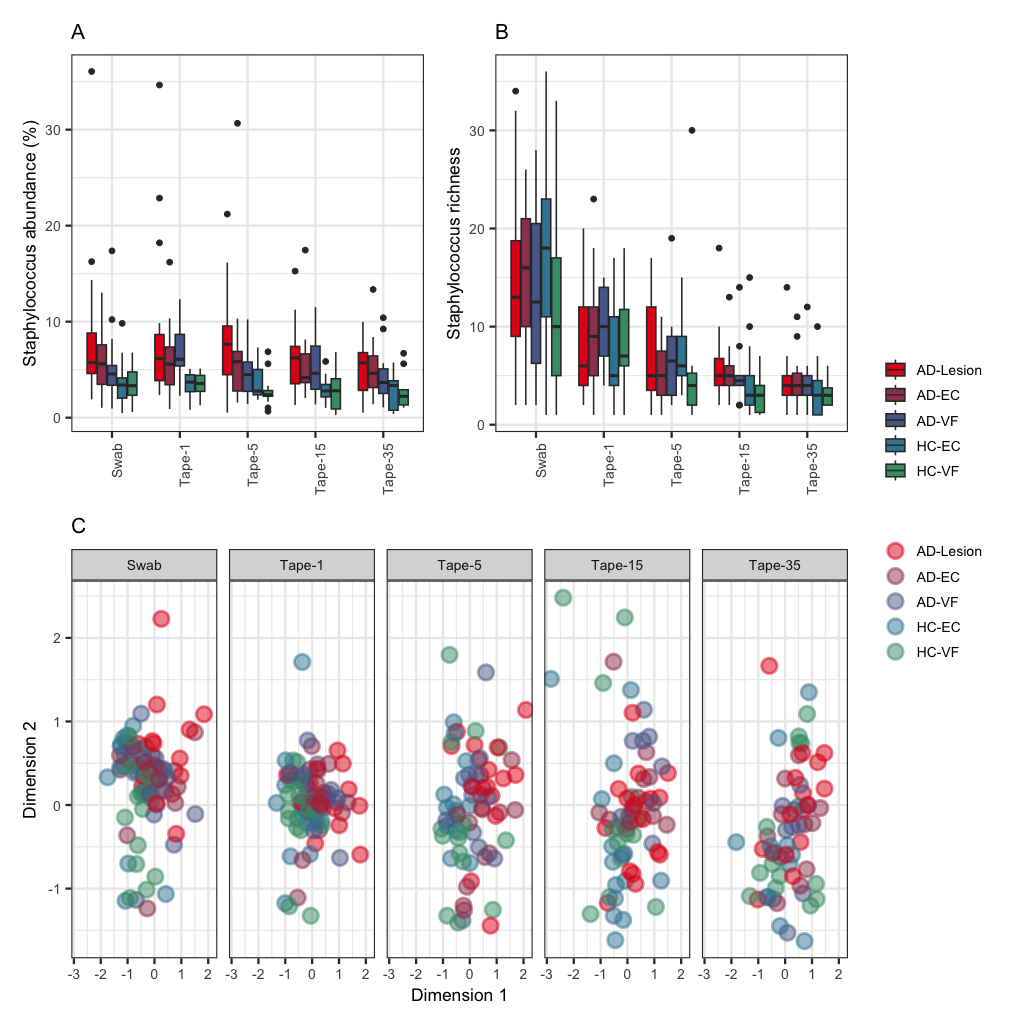


**Figure S7** The *Staphylococcus* community was extracted from the bacterial dataset. Differences in (A) *Staphylococcus* relative abundance, (B) richness and (C) community composition were visualised to investigate for differences between skin types.
